# Pretreatment Patient-reported Overall Health: A Prognostic Factor for Early Overall Mortality After Primary Curative Treatment of Prostate Cancer

**DOI:** 10.1016/j.euros.2024.03.005

**Published:** 2024-03-23

**Authors:** Sophie D. Fosså, Anne Holck Storås, Kirsti Aas, Tom Børge Johannesen, Ylva Maria Gjelsvik, Tor Å. Myklebust

**Affiliations:** a Department of Oncology, Oslo University Hospital, University of Oslo, Oslo, Norway; b Institute of Clinical Medicine, University of Oslo, Oslo, Norway; c Department of Research, Cancer Registry of Norway, Oslo, Norway; d Department of Urology, Akershus University Hospital, Lørenskog, Norway; e Department of Registration, Cancer Registry of Norway, Oslo, Norway; f Department of Research and Innovation, Møre and Romsdal Hospital Trust, Ålesund, Norway

**Keywords:** Prostate cancer, Curative treatment, Pretreatment patient-reported health, Treatment choice, Short-term overall survival

## Abstract

**Background and objective:**

Registry-based studies for prostate cancer (PCa) document higher overall mortality (OM) after high-dose radiotherapy (RT) than after radical prostatectomy (RP). Our aim was to explore the association between pretreatment patient-reported health (“OverallHealth”: OH) and curative treatment type, and the impact on early OM.

**Methods:**

New PCa patients registered between 2017 and 2019 in the Cancer Registry of Norway (*n* = 1949) completed the European Organisation for Research and Treatment of Cancer Quality-of-Life Core 30 (QLQ-C30) questionnaire before RP (*n* = 592) or RT (*n* = 610) or after allocation to active surveillance (AS; *n* = 747). We dichotomised the QLQ-C30 summary score to classify patients with un-impaired versus impaired OH*.* Standard univariable and multivariable analyses with treatment type or OM as the outcome were conducted. The mean observation time was 4.7 years (standard deviation 1.0). Statistical significance was set at *p* < 0.05.

**Key findings and limitations:**

Impaired OH was more frequent in the RT group (38%) than in the RP (25%) or AS (28%) group (*p* < 0.001). Higher age, higher risk group, and impaired OH increased the probability of undergoinRT rather than RP (*p* < 0.001). Impaired OH was associated with a twofold higher early OM rate in the RT group (16% vs 8%; *p* = 0.009) and fourfold higher OM rate in the AS group (13% vs 3%; *p* < 0.001). These findings remained significant in Cox regression analyses controlled for age and risk group. After RP, only locally advanced high-risk tumours were significantly associated with OM. Unknown psychometrics for the OH variable is the main study limitation.

**Conclusions and clinical implications:**

Pretreatment patient-reported impaired OH, measured as the QLQ-C30 summary score, was positively associated with allocation to RT or AS and is a prognostic factor for early OM. Before allocation to RT or AS, elderly patients with PCa should be screened and treated for health problems that can be remedied. Future studies should determine the psychometrics of the QLQ-C30 summary score in comparison to established frailty screening instruments.

**Patient summary:**

Patient-reported scores reflecting their overall health can help in choosing curative treatment for prostate cancer and are associated with survival during the first 5 years after treatment.

## Take Home Message

Patient-reported overall health before treatment can guide allocation to the type of curative treatment for prostate cancer. Impaired overall health significantly increases early overall mortality after radiotherapy and active surveillance. In daily practice, better patient selection for radiotherapy or active surveillance is needed.

## 1 Introduction

Radical prostatectomy (RP), high-dose radiotherapy (RT; equivalent dose in 2-Gy fractions: ≥74 Gy), and active surveillance (AS) are curative treatment modalities for patients with nonmetastatic prostate cancer (PCa) with a life expectancy of ≥10 yr [1]. In two randomised trials, 10-yr PCa-specific mortality and overall mortality (OM) were independent of the treatment type [2], [3]. By contrast, registry-based analyses revealed worse OM afterRT in comparison to RP [4], [5], [6], [7], [8] as comorbidity is commonly more frequent in RT than in RP cohorts [9], [10]. Comorbidity is not routinely documented in national cancer registries [11]. However, in the Cancer Registry of Norway (CRN), the physician-rated Eastern Cooperative Oncology Group performance status (ECOG PS) [12] at the time of diagnosis is available and serves as a proxy for comorbidity. Frailty is recognised as an additional risk factor for adverse outcomes after curative treatment for PCa, at least in patients aged ≥70 years. Screening tools for frailty have been developed [13].

In recent years, patient-reported treatment-related changes in functions, symptoms, and health-related quality of life (QoL) have been integrated into oncological practice [14], [15]. The summary score from the European Organisation for Research and Treatment of Cancer Quality-of-Life Questionnaire (QLQ-C30) [16] reflects a patient’s overall evaluation of physical, mental and social health. It has been shown that this summary score, in the current study called OverallHealth (OH) is a prognostic factor in cancer patients [17], [18], [19]. For patients with newly diagnosed PCa, the clinical relevance of OH assessed before treatment has not been analysed in relation to the final treatment choice or as prognostic factor for OM.

Our observational explorative study, which also had access to normative data, therefore asked the following questions:

- What is the association between pretreatment OH and the choice of curative treatment modality for patients with PCa?
- Is OH associated with early OM, defined as death within 5 years after diagnosis, thus representing a prognostic factor?

As a secondary objective, we investigated the clinical relevance of EGOG PS for treatment allocation and OM.

## 2 Patients and methods

### 2.1 Data sources

#### 2.1.1 Patients

For each man with a new diagnosis of nonmetastatic PCa (Diagnosis; 2017-2019), the following data were extracted from the CRN: date of birth, risk group, date of RP or of initiation of RT ECOG PS, and date and cause of death, if applicable. The clinical registration form provided information about the treatment intention: noncurative versus curative, with AS distinguished from watchful waiting (WW). In 2017, the CRN started a three-round questionnaire-based survey that should capture changes in QoL in PCa patients during the first post-diagnosis years. Among other instruments, participants completed version 3 of the QLQ-C30.

Evaluable patients fulfilled all of the following criteria:

- Completion of all the QLQ-C30 scales within 3 months after diagnosis and before the date of RP or initiation of RT. The 3-months limit minimised the impact of pre-RT neoadjuvant hormone therapy.
- Curative treatment with RP oRT during the first year after diagnosis, or allocation to AS without RP or RT during the first year.
- Documentation of ECOG PS. Owing to the small number of patients with ECOG PS 2, this category was excluded.

Evaluable patients were stratified according to the type of curative treatment, PCa risk group, and age at diagnosis (<65 yr, 65-74 yr, or ≥75 years).

#### 2.1.2 Control subjects

The CRN also established a control group by frequency matching on age. Control subjects were men from the general population registry without a PCa diagnosis (*n* = 10 966), of whom 3258 completed the same questionnaire as the PCa patients after omitting PCa-specific questions.

### 2.2 QLQ-C30 scores

QLQ-C30 responses were transformed to scale scores (from 0 to 100) as recommended in the scoring manual [20]. The summary score reflecting OH was calculated after omitting items assessing diarrhoea, constipation, and appetite loss [21]. Higher scale scores for global QoL and OH reflect better function, whereas lower scores for the symptom scales reflect less bothersome symptoms.

For each study group and for each QLQ-C30 scale we calculated the proportion of patients with impaired versus un-impaired scale scores. Impaired functions or symptoms were identified as scores below (functional scores) or above (symptom scores) the scale-specific cutoff score that separated the most unfavourable tertile from the two more favourable tertiles in the control group.

### 2.3 Statistical analysis

Results for continuous variables are presented as means or medians. Percentages were computed for categorical variables. Independent t tests and χ^2^ tests were used to explore the statistical significance of differences, and Cohen’s d was calculated for between-group differences in the scale scores. The Kaplan-Meier method was used to estimate OM, with a log-rank test for assessment of between-group differences. The observation time ranged from the date of PCa diagnosis to March 31, 2023. Multivariable analyses included age, tumour risk group and OH (ECOG PS in the secondary analyses) as the independent variable, with treatment modality or OM as the outcome. Cox proportional hazards were estimated for OM, and adjusted survival curves were plotted for the model for specific covariate patterns. Statistical significance was set to the standard *p* < 0.05. Cohen’s d > 0.20 was defined as clinically significant. Analyses were performed using SPSS v29 and Stata v18.1. No age adjustment was performed for the QLQ-C30 scores as Cohen’s d was <0.20 for all score differences between the PCa cohort and the control group except for the pain scale (Supplementary Table 1).

### 2.4 Study ethics

All patients provided written consent. The Regional South-East Committee for Medical and Health Research Ethics approved the study (reference 2015/1294).

## 3 Results

### 3.1 PCa patients

Our selection criteria were met by 1949 patients (Table 1 and Supplementary Fig. 1). In comparison to the RP group (*n* = 592), men in the RT group (*n* = 610; *p* < 0.001) and in the AS group (*n* = 747; *p* = 0.035) were older. In the RT group, the proportion of patients with locally advanced high-risk PCa was twice as high and the proportion with ECOG PS 1 was three times as high as in the RP group. During the observation period (mean 4.7 ± 1.0 yr), 101patients died.

**Table 1:** Baseline characteristics of the study cohorts:

	RP (*n* = 592)	RT (*n* = 610)	AS (*n* = 747)	All PCa (*n* = 1949)	Controls (*n* = 3258)
Age (yr) a	66 (61-70)	72 (69-75)	68 (62-71)	68 (69-73)	69 (63-74)
Age category, *n* (%)					
≤64 yr	267 (45)	68 (11)	304 (41)	639 (33)	1041 (32)
65-74 yr	296 (50)	341 (56)	380 (51)	1017 (52)	1473 (45)
≥75 yr	29 (5)	201 (33)	63 (8)	293 (15)	744 (23)
Education level, *n* (%)					
<12 yr	277 (47)	323 (54)	369 (50)	969 (50)	1736 (53)
≥12 yr	310 (53)	278 (46)	366 (50)	954 (50)	1493 (47)
Data missing	5	9	12	26	29
Risk group, *n* (%)					-
Low risk	27 (5)	8 (1)	509 (68)	544 (28)	
Intermediate risk	247 (42)	140 (23)	238 (32)	628 (32)	
High risk, localised	187 (32)	167 (28)	0	354 (18)	
High risk, locally advanced	128 (22)	292 (48)	0	420 (22)	
Data missing	3	3	0	6	
Previous cancer ≤5 yr, *n* (%)	16 (2)	25 (4)	31 (4)	72 (4)	-
ECOG PS, *n* (%)					-
0	555 (94)	493 (81)	679 (91)	1727 (89)	
1	37 (6)	117 (19)	68 (9)	222 (11)	
Observation time (yr) a	5.0 (0.4-6.2)	4.8 (0.6-6.2)	4.9 (0.2-6.2)	4.9 (0.2-6.2)	-
Deaths, *n* (%)	18 (3.0)	45 (7.4)	38 (5.1)	101 (5.2)	
PCa	0	6	2	8	
Other cancer	5	12	12	29	-
Cardiovascular disease	4	8	9	21	
Other/unknown	9	19	15	43	

AS = Active Surveillance: ECOG PS=Eastern Cooperative ONcology Grouå perforemance status, PCa= Prostate Cancer, RP=Radical Prostatectomy, RT=Radiation Therapy.

a Median (interquartile range).

### 3.2 QLQ-C30 scores

**Fig. 1:**
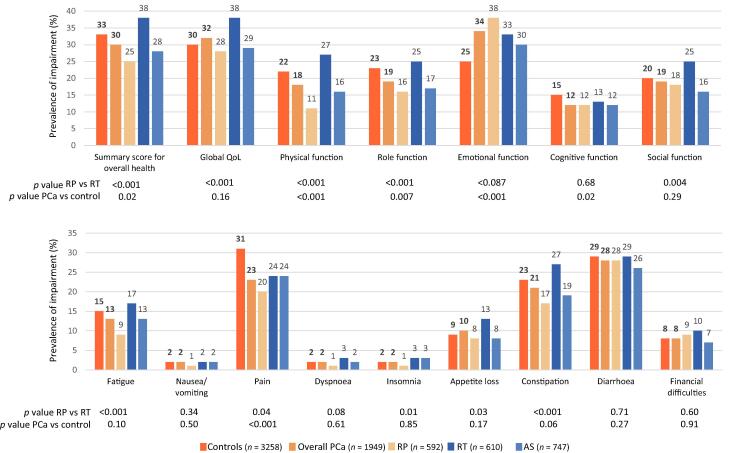
Prevalence of impaired OverallHealth within the three treatment groups and the controll group. AS = Active Surveillance; PCa = Prostate Cancer; RP = Radical Prostatectomy; RT = Radiation Therapy.

Between-group differences for the QLQ-C30 scale scores were small (Supplementary Fig. 2), but became more evident on comparison of the proportions of men with impairment for each scale by group (Fig. 1). The largest differences between the RP and the RTgroup (*p* < 0.001) were observed for impaired OH (25% vs 38%) and impaired physical function (11% vs 27%). The prevalence of fatigue and pain was also higher in the RAD group than in the RP group. The impairment rates for the AS group were intermediate between those for the RP group and the RAD group. Interestingly, the prevalence of impaired OH was greater in the control group than in the overall PCa group (33% vs 30%; *p* < 0.02), with the greatest difference between the control and RP groups (33% vs 25%; *p* < 0.001). Pain was more frequent among control subjects than among PCa patients, with opposite findings for emotional distress.

### 3.3 Treatment allocation

Impaired OH doubled the odds of being allocated toRT in comparison to RP (OR 2.15, 95% CI 1.60-2.89; *p* < 0.001; Table 2). In comparison to patients aged <65 yr, those aged ≥75 yr had more than 20-fold higher odds of receiving RT. Patients with intermediate-risk tumours were rarely assigned to AS (OR 0.04, 95% CI 0.03-0.06; *p* < 0.001).

**Table 2:** Odds of being allocated to treatment

Parameter	RP versus RT		RP/RT versus AS	
	OR (95% CI)	*p* value	OR (95% CI)	*p* value
Age (yr)				
<65	Reference	**<0.001**	Reference	0.616
65-74	5.23 (3.75-7.28)	<0.001	1.11 (0.80-1.53)	0.531
≥75	27.35 (16.70-44.78)	0.001	1.27 (0.78-2.06)	0.343
Risk group				
Low/intermediate risk	Reference	**<0.001**		
High risk, local	1.48 (1.07-2.05)	0.017		
High risk, locally advanced	3.83 (2.77-5.30	<0.001		
Intermediate risk vs low risk			0.04 (0.03-0.06)	**<0.001**
Impaired OH (vs unimpaired)	2.15 (1.60-2.89)	**<0.001**	0.96 (0.69-1.32)	0.781

CI = confidence interval; OH = overall health; OR = odds ratio; RP = Radical Prostatectomy; RT = high-dose Radiation therapy.

### 3.4 Overall mortality

**Fig. 2:**
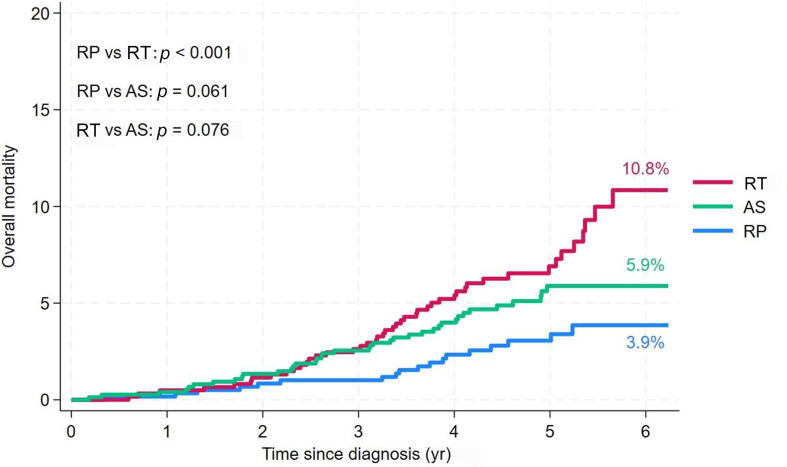
Kaplan-Meier plot of overall mortality by treatment group. The *p* values are for log-rank tests. AS = active surveillance; RP = radical prostatectomy; RT = radiation therapy.

**Fig. 3:**
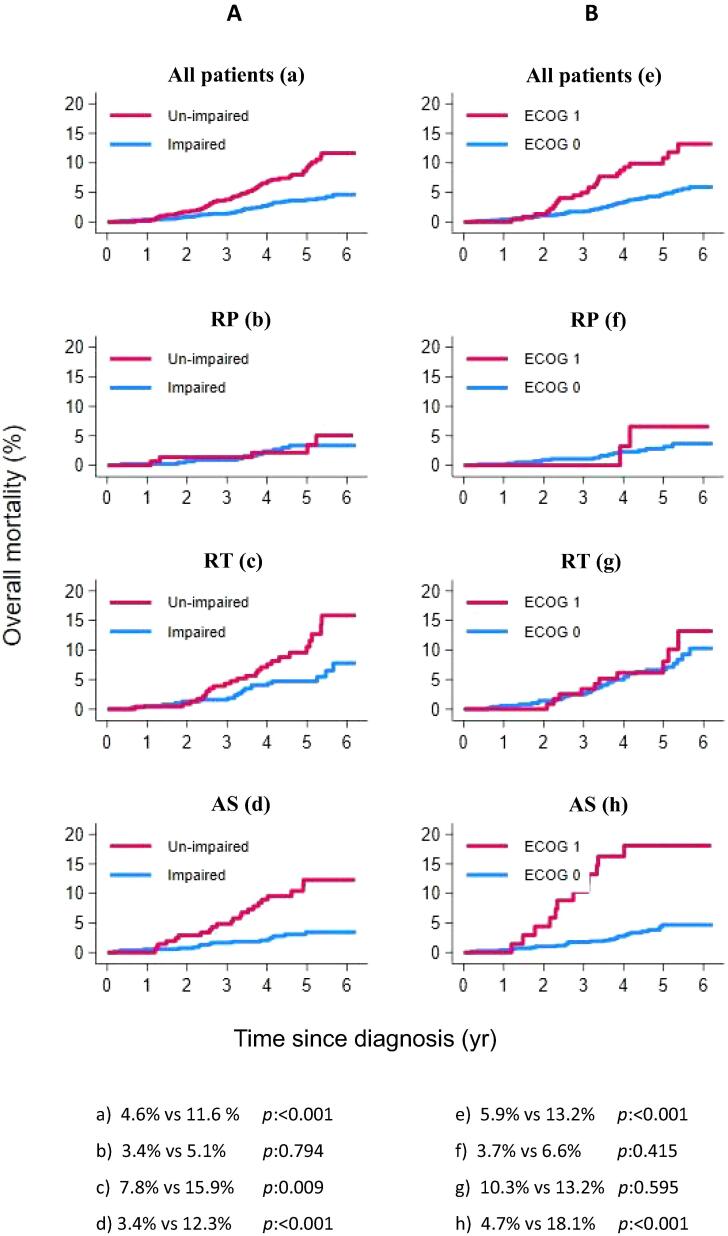
Kaplan-Meier plots of overall mortality in the overall prostate cancer cohort and treatment groups stratified by (A) un-impaired versus impaired Overall Health and (B) by the Eastern Cooperative Oncology Group (ECOG) performance status (0 vs 1). The *p* values are for log-rank tests. AS = Active Surveillance; RP = Radical Prostatectomy; RT = Radiation Therapy.

At the end of the observation period, the highest OM rate was observed in the RTgroup (10.8%; Fig. 2). Impaired OH was significantly associated with worse OM in the entire PCa cohort and in the RT and AS groups, but not in the RP group (Fig. 3A). These findings were confirmed in Cox regression analyses (Table 3). In theRT group, impaired OH was the only factor significantly associated with the risk of OM (HR 2.28, 95% CI 1.28-4.16; *p* = 0.001), although there was also a strong nonsignificant association between older age and OM after RT.

**Table 3:** Cox regression analyses for overall mortality as the outcome and age, risk group, and OH as independent variables

Parameter	Radical Prostatectomy		Radiation Therapy		Active Surveillance		Overall prostate cancer	
	HR (95% CI)	*p* value	HR (95% CI)	*p* value	HR (95% CI)	*p* value	HR (95% CI)	*p* value
Age (yr)								
<65	Reference	0.987	Reference	0.143	Reference	**0.002**	Reference	**<0.001**
65-74	0.95 (0.37-2.48)	0.922	6.27 (0.85-46.49)	0.072	2.99 (1.21-7.36)	0.017	2.55 (1.44-4.51)	0.001
≥75	1.12 (0.14-8.94)	0.918	7.49 (1.00-56.26)	0.050	6.94 (2.41-20.04)	<0.001	4.06 (2.14-7.71)	<0.001
Risk group								
LR/IR	Reference	**0.016**	Reference	0.907			Reference	
High, local	2.98 (0.74-11.90)	0.123	0.98 (0.45-2.12)	0.959			1.06 (0.63-1.77)	0.875
High, LA	6.55 (1.77-24.30)	0.005	0.87 (0.42-1.79)	0.697			1.13 (0.71-1.82)	0.837
Intermediate risk (vs LR)					1.37 (0.71-2.66)	0.349		0.606
Unimpaired OH (vs unimpaired)	1.02 (0.36-2.87)	0.97	2.31 (1.28-4.16)	**0.005**	3.69 (1.94-7.04)	**<0.001**	2.49 (1.68-3.68)	**<0.001**

CI = confidence interval; HR = hazard ratio; IR = intermediate risk; LA = locally advanced; LR = low risk; OH = OverallHealth.

After stratification by PCa risk group, impaired OH remained an independent prognostic factor for OM in the RT and AS groups (Supplementary Fig. 3). In the RP group, only the presence of a locally advanced high-risk tumour was associated with OM (Table 3).

### 3.5 ECOG PS

When we replaced the OH variable with ECOG PS, we observed similar findings regarding treatment allocation and OM (data not shown). However, ECOG PS was not associated with OM in the RT group (Supplementary Table 2 and Fig. 3B).

## 4 Discussion

In this real-world study of curative treatment of PCa, the rate of patient-reported impaired OH was doubled in the RT groupcompared to the prevalence of impaired OH in the RP group. Impaired OH significantly increased the risk of early OM (twofold in the RT group and threefold in the AS group). In the RP group, only the presence of a locally advanced high-risk tumour was significantly associated with early OM.

Similar results regarding treatment allocation and OM were observed for all patients when OH was replaced by ECOG PS. However, in the RT group ECOG PS was not associated with early OM.

### 4.1 Dichotomisation of QLQ-C30 scale scores

We did not use published QLQ-C30 threshold scores indicating clinical importance [21]*.* These values were established in cancer patients whose health was probably impaired by their advanced malignancy and/or ongoing or recent treatment. Our results show that pretreatment QLQ-C30 scores reported by our PCa patients, and thus the clinically important threshold values, were similar to and sometimes even higher than those reported by the men in the control group. In our view, these findings reflect the positive selection of patients with PCa for curative treatment, particularly for RP. Following other investigators [18], [22], we dichotomised our OH score. The cutoff was the OH score that separated the two upper tertiles (un-impaired OH) from the third tertile (impaired OH) in the control group.

### 4.2 OverallHealth

Our mean pre-RP summary score (90 points) is comparable to a mean pre-RP score reported from Japan (>95 points) [23] and similar to a pre-RP score for Dutch patients (93 points), but is higher than a German score (88 points) [24]. These high mean scores reflect the favourable selection of RP patients. The highest impaired OH rate in our study was observed in the RT group. This agrees with results from observational registry-based studies [6], [7], [8], [9] indicating higher prevalence of pre-RT comorbidity assessed in terms of the physician -established Charlson comorbidity index (CCI) [25]. Notably, while the CCI reflects previously established diagnoses, the patient-reported OH variable emphasises current functions and symptoms, probably mirroring frailty better than the CCI.

Our findings regarding treatment allocation and impaired OH reflect a common referral practice for selecting Norwegian patients with PCa for RT. Pretreatment screening using tests of frailty or geriatric assessments [1,13]are not routinely performed during the urologist-led diagnostic period. The health status and suitability of patients for whom RP is being considered are routinely categorised using the American Society of Anesthesiologists system [26]. Patients who are not candidates for surgery because of high age and/or poor health are then offered RT if it is considered that curative treatment will be beneficial for them. When these patients subsequently meet the radiation oncologist they are mentally primed to expect curative treatment and they are not prepared to accept the non-curative WW strategy. In this situation, psychological considerations may lead to RT initiation in frail patients or in those aged ≥85 years. Importantly,even the AS strategy requires a life expectancy of ≥10 years to allow for subsequent curative local treatment. Whether our patients aged ≥85 years met this requirement appears at least questionable. The above patient selection process for RP and the referral practice described also explains the low prevalence of impaired OH in our RP group. We can only speculate to what degree similar referral routines are followed in other countries.

### 4.3 Overall mortality

As in other real-world studies [4], [7], our OM rate was significantly higher after RT than after RP. Furthermore, in the RT group, impaired OH was the only factor significantly associated with early OM after treatment. Our findings highlight the need for better identification of patients with high risk of early OM after RT. Our results show that patient-reported OH can contribute to identification of men with impaired health who need successful rehabilitation before allocation to curative PCa treatment, at least before initiation of RT. Furthermore, the oldest patients eligible for RP or RT should ideally be included in studies exploring the outcomes of different treatment modalities. For example, SPCG-19 (ClinicalTrials.gov NCT05448547) is investigating outcomes for curative local intervention in comparison to WW. In our AS group the significant impact of older age in contributing to the effect of impaired OH calls for better distinction between a planned AS strategy and WW. The more careful patient selection for RP and the relatively short observation time may explain why impaired OH was not associated with OM in the RP group.

Screening for frailty using validated tools and the establishment of geriatric profiles [1], [10], [13], [27] are recommended tasks before curative treatment of elderly patients with PCa. It is anticipated that identification of health problems that can be remedied via successful rehabilitation reduce treatment-related adverse health outcomes, including early OM. Our results indicate that this strategy is particularly important before referral for RT, which, in contrast to AS, is associated with bothersome side effects. Multidisciplinary teams (MDTs) can support treatment decisions [28], [29]. However, it is currently unknown to what degree the recommended resource-requiring tools (geriatric assessment, MDTs) are used in daily practice and how effectively they reduce early OM for patients who have undergone curative treatment for PCa. Our results suggest that self-reported OH assessed before RT or allocation to AS can serve as a screening tool to reduce early OM. In clinical practice, this assessment can be implemented before a consultation if the patient responds to a digital version of the QLQ-C30 electronically connected to a calculation program, Future studies should compare the usefulness and validity of the OH variable with information obtained via recommended screening tools for frailty and/or by established comorbidity indices..

### 4.4 ECOG PS

Our results confirm previous findings on the prognostic significance of ECOG PS in large cohorts [5]. However, the lack of significance for ECOG PS as a prognostic factor in the RT group warrants the use of more sensitive tools before RT.

### 4.5 Limitations

First, owing to the stringent selection criteria, our study population includes only 16% of all patients with nonmetastatic PCa registered in the CRN from 2017 to 2019. Second, self-reported OH has not yet been compared with objective comorbidity data. Third, in this national registry.-based study the possibility of interobserver variability for some key variables i cannot be excluded. In particular, separation of AS from WW appears to be unreliable in clinical practice, supporting the need for combination of AS and WW in registry-based studies. The first real-world documentation of the clinical relevance of patient-reported OH before curative PCa treatment is the strength of this proof-of-evidence study.

## 5 Conclusions

Patient-reported OH before treatment can assist the allocation of patients with PCa to curative treatment modalities. Impaired OH significantly increased the risk of early OM after RT or AS. Early OM is probably reduced by better selection of patients for curative treatment of PCa on the basis of their OH, particularly if RT or AS is being considered. Elderly patients should be screened for impaired OH before treatment and should, if necessary, be referred for rehabilitation. The patient-reported QLQ-C30 summary score seems to represent an easily available tool for screening for impaired OH, although future psychometric validation is needed.

  ***Author contributions***: Sophie D. Fosså had full access to all the data in the study and takes responsibility for the integrity of the data and the accuracy of the data analysis.

  *Study concept and design*: Fosså, Storås, Myklebust.

*Acquisition of data*: Johannesen, Gjelsvik.

*Analysis and interpretation of data*: Fosså, Myklebust.

*Drafting of the manuscript*: Fosså, Storås, Aas.

*Critical revision of the manuscript for important intellectual content*: All authors.

*Statistical analysis*: Fosså, Myklebust.

*Obtaining funding*: Fosså, Johannesen.

*Administrative, technical, or material support*: Fosså.

*Supervision*: Fosså.

*Other*: None.

  ***Financial disclosures:*** Sophie D. Fosså certifies that all conflicts of interest, including specific financial interests and relationships and affiliations relevant to the subject matter or materials discussed in the manuscript (eg, employment/affiliation, grants or funding, consultancies, honoraria, stock ownership or options, expert testimony, royalties, or patents filed, received, or pending), are the following: None.

  ***Funding/Support and role of the sponsor*:** This work was supported by Movember True NTH Global Registry (project 17016001, Norwegian Cancer Society and Norwegian Radium Hospital Foundation, grant number 335007). The sponsor played a role in data analysis.

  ***Data sharing statement*:** Data are not available to other researchers. The study used registry or institutional database data routinely collected for patients.
