# Supplementary material for: Pretreatment Patient-reported Overall Health: A Prognostic Factor for Early Overall Mortality After Primary Curative Treatment of Prostate Cancer
Source: Eur Urol Open Sci. 2024 Mar 23;63:62–70. doi: 10.1016/j.euros.2024.03.005 (PMC10979064; doi:10.1016/j.euros.2024.03.005)
Supplement: Supplementary data 1 [file mmc1.pdf]

Suppl. Figure 1: Flow Chart

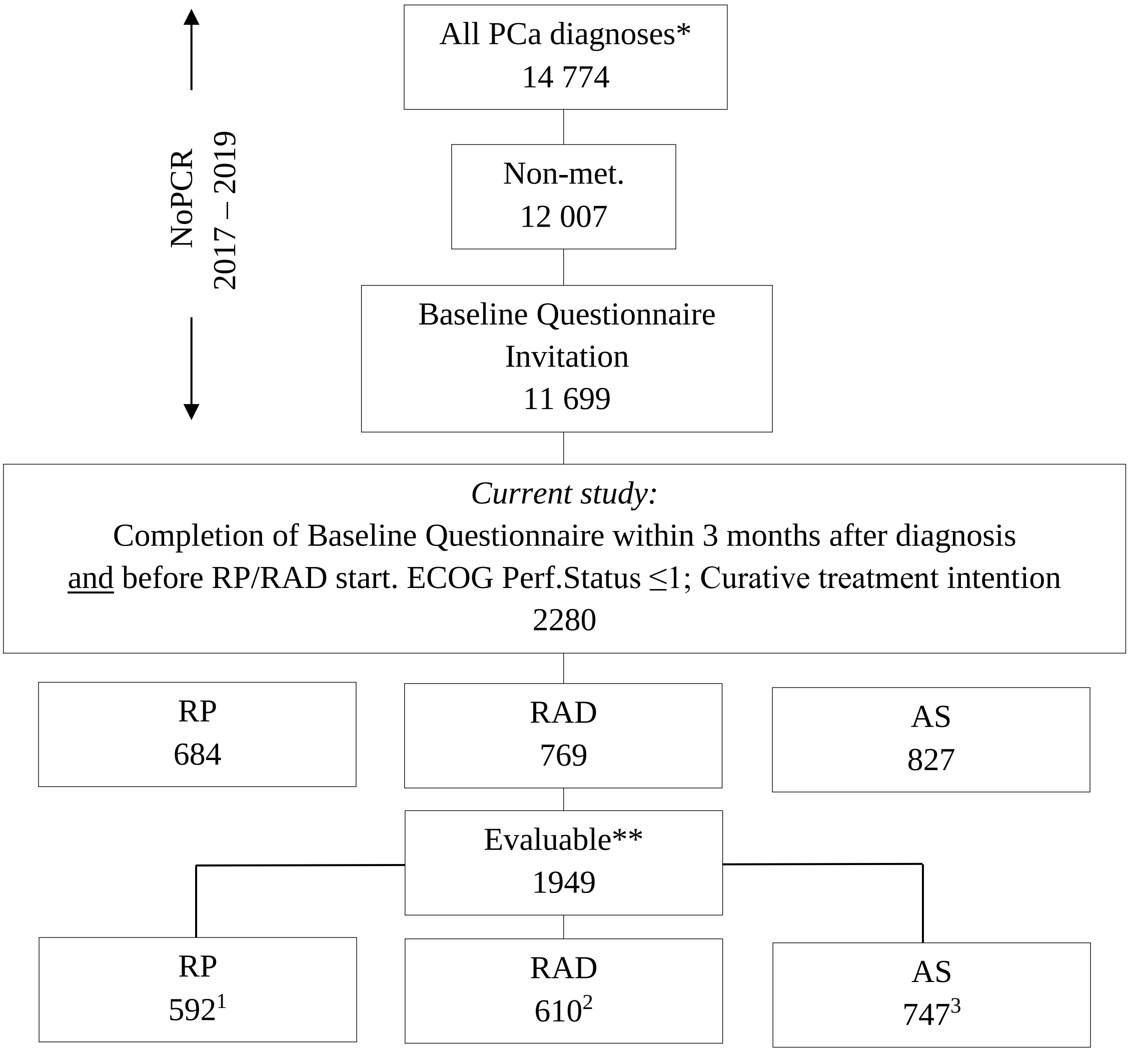

*After exclusion of 338 patients with PCa diagnosis based on autopsy (n: 36) or Cystoprostatectomy (n: 302)

**Valid EORTC QLQ C-30

[1] RP within 1 year after diagnosis
[2] Start of RAD within 1 year after diagnosis
[3] No RP or RAD within 1 year after diagnosis
