# Supplementary material for: Pretreatment Patient-reported Overall Health: A Prognostic Factor for Early Overall Mortality After Primary Curative Treatment of Prostate Cancer
Source: Eur Urol Open Sci. 2024 Mar 23;63:62–70. doi: 10.1016/j.euros.2024.03.005 (PMC10979064; doi:10.1016/j.euros.2024.03.005)

Suppl.Figure 1: QlQ-C30 scale scores and Cohen's d comparing a) RP versus RAD or b) all PCa patients versus Norms

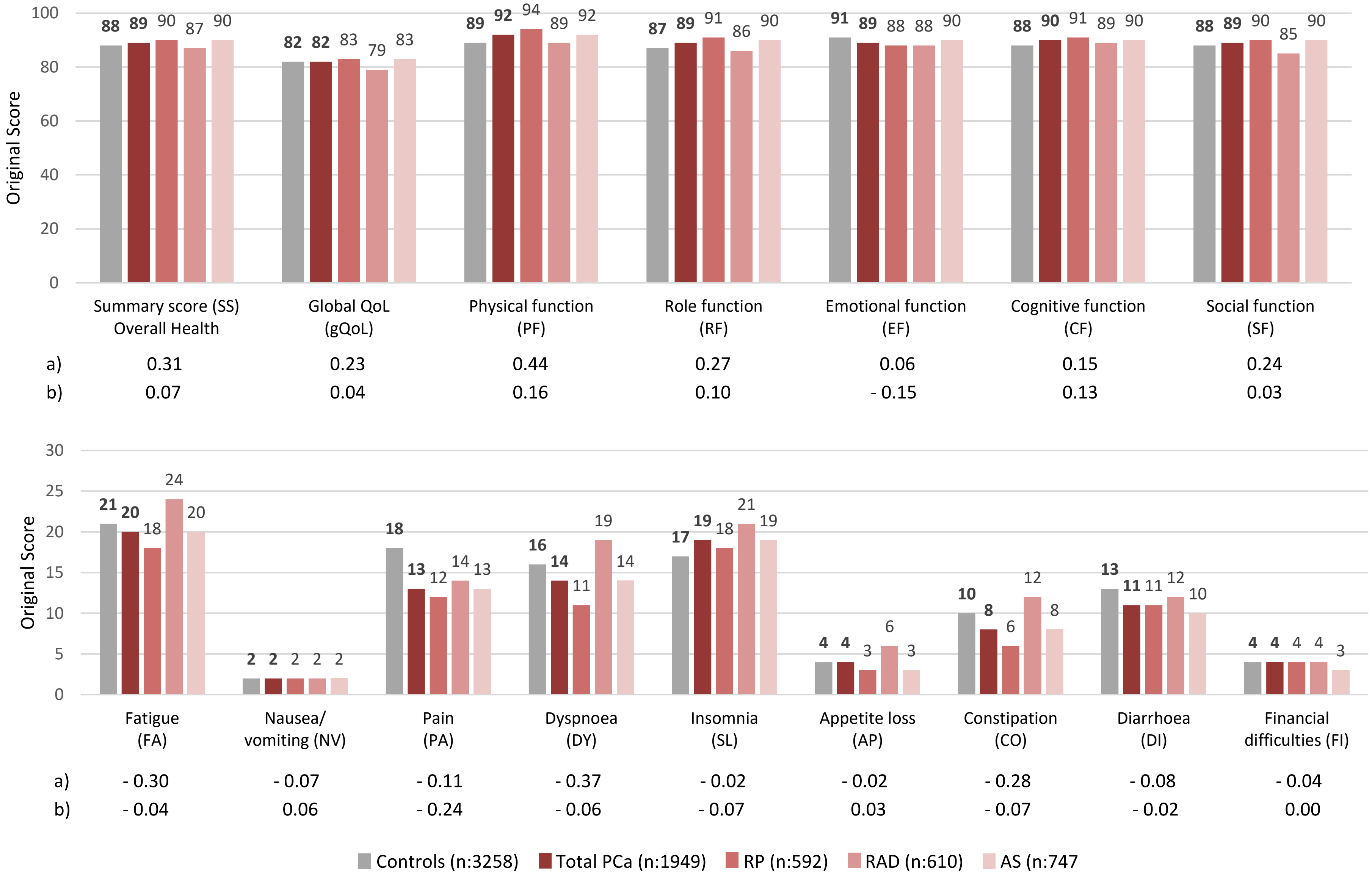

Supplement: Supplementary data 2 [file mmc2.pdf]
