# Supplementary material for: Pretreatment Patient-reported Overall Health: A Prognostic Factor for Early Overall Mortality After Primary Curative Treatment of Prostate Cancer
Source: Eur Urol Open Sci. 2024 Mar 23;63:62–70. doi: 10.1016/j.euros.2024.03.005 (PMC10979064; doi:10.1016/j.euros.2024.03.005)

Figure 3 . Predicted Overall mortality stratified for risk groups : Un-impaired versus Impaired OverallHealth

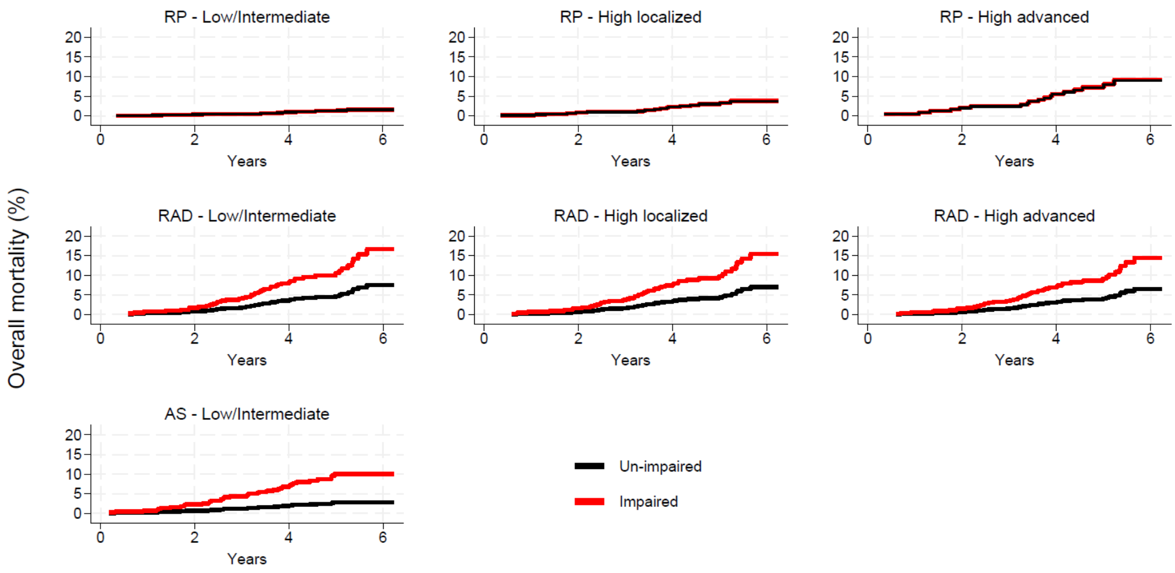

Supplement: Supplementary data 3 [file mmc3.pdf]
