# Supplementary material for: Pretreatment Patient-reported Overall Health: A Prognostic Factor for Early Overall Mortality After Primary Curative Treatment of Prostate Cancer
Source: Eur Urol Open Sci. 2024 Mar 23;63:62–70. doi: 10.1016/j.euros.2024.03.005 (PMC10979064; doi:10.1016/j.euros.2024.03.005)
Supplement: Supplementary data 4 [file mmc4.docx]

|  | **Norms** | | **PCa patients** | |
| --- | --- | --- | --- | --- |
|  | **Mean** | **SD** | **Mean** | **SD** |
| **Function subscales** |  |  |  |  |
| Physical function (PF) | 89.2 | 17.0 | 91.7 | 14.4 |
| Role function (RF) | 86.8 | 23.3 | 89.0 | 20.4 |
| Emotional function (EF) | 91.2 | 14.8 | 88.9 | 15.2 |
| Cognitive function (CF) | 87.8 | 15.8 | 89.8 | 14.9 |
| Social function (SF) | 88.2 | 20.8 | 88.7 | 18.4 |
| Global Quality of life (gQoL)  Summary score (OverallHealth) | 82.3  88.0 | 18.9  12.2 | 81.6  88.9 | 17.7  11.7 |
| **Symptoms subscales/Item** |  |  |  |  |
| Fatigue (FA) | 21.0 | 20.5 | 20.2 | 19.8 |
| Nausea/Vomiting (NV) | 1.7 | 6.1 | 2.0 | 8.0 |
| Pain (PA)* | 18.2 | 22.8 | 12.9 | 19.9 |
| Dyspnea (DY) | 15.6 | 23.5 | 14.2 | 22.8 |
| Insomnia (SL) | 17.4 | 24.7 | 19.1 | 24.7 |
| Appetite loss (AP) | 3.6 | 12.5 | 4.0 | 13.1 |
| Constipation (CO) | 10.0 | 20.0 | 8.6 | 18.5 |
| Diarrhea (DI) | 11.3 | 19.4 | 10.9 | 19.3 |
| Financial difficulties (FI) | 3.8 | 14.2 | 3.8 | 14.3 |

**Suppl.Table 1: QLQ -C30 scale scores in PCa patients (n:1949) and Norms ( n: 3258)**

*Cohen’s d: -0.24 ; all other differences Cohen’s d : <0.20
