# Supplementary material for: Pretreatment Patient-reported Overall Health: A Prognostic Factor for Early Overall Mortality After Primary Curative Treatment of Prostate Cancer
Source: Eur Urol Open Sci. 2024 Mar 23;63:62–70. doi: 10.1016/j.euros.2024.03.005 (PMC10979064; doi:10.1016/j.euros.2024.03.005)
Supplement: Supplementary data 5 [file mmc5.docx]

**Suppl. Table 2:** Cox regression analyses with Overall Mortality being the outcome and Age, Risk group and *ECOG performance statu*s as independent variables : All patients and stratifications for treatment group

|  | **RP** | | | **RAD** | | | **AS** | | | **All** | | |
| --- | --- | --- | --- | --- | --- | --- | --- | --- | --- | --- | --- | --- |
|  | **HR** | **95% CI** | **p** | **HR** | **95% CI** | **p** | **HR** | **95% CI** | **p** | **HR** | **95% CI** | **p** |
| **AGE**  <65  65-74  >75 | Ref.  0.88  1.09 | 0.33; 2.36  0.14; 8.75 | 0.955  0.791  0.935 | Ref.  5.36  6.52 | 0.73; 39.65  0.87; 48.95 | 0.180  0.100  0.068 | Ref.  2.70  4.33 | 1.09; 6.72  1.40; 13.32 | **0.034**  0.033  <0.011 | Ref.  2.29  3.56 | 1.29; 4.07  1.86; 6.85 | **<0.001**  0.005  0.001 |
| **Risk Group**  A: Low/Intermed.  High local  High local adv.  B: Low (Ref.)  Intermediate | Ref.  3.09  6.58 | 0.80; 12.44  1.78; 24.36 | **0.016**  0.112  0.005 | Ref.  1.03  0.86 | 0.47; 2.23  0.42; 1.78 | 0.865  0.949  0.687 | Ref.  1.26 | 0.65; 2.45 | 0.492 | 1.15  1.16 | 0.68; 1.93  0.73; 1.86 | 0.774  0.604  0.503 |
| **ECOG-PS**  0  1 | Ref.  1.89 | 0.41; 8.79 | 0.415 | Ref.  1.18 | 0.58; 2.41 | 0.643 | Ref.  3.32 | 1.58; 6.99 | **0.002** | Ref.  1.95 | 1.21; 3.12 | **0.006** |
